# Characterizing the visualization design space of distant and close reading of poetic rhythm

**DOI:** 10.3389/fdata.2023.1167708

**Published:** 2023-06-06

**Authors:** Alejandro Benito-Santos, Salvador Muñoz, Roberto Therón Sánchez, Francisco J. García Peñalvo

**Affiliations:** ^1^Digital Humanities Innovation Lab (LINHD), National University of Distance Education, Madrid, Spain; ^2^Research Group on Interaction and e-Learning (GRIAL), University of Salamanca, Salamanca, Spain

**Keywords:** poetry, text visualization, digital humanities, cultural collections, automatic scansion analysis, natural language processing

## Abstract

Metrical and rhythmical poetry analysis is founded on the systematic statistical analysis and comparison of sonic devices (e.g., rhythmic patterns) that emerge from a combination of pre-established aesthetic and structural rules and the poet's abilities and creative genius to convey a given message adhering to the said constraints. These rhythmical patterns, which have been traditionally obtained by means of a careful close reading of the poems, in a process known as “scansion,” can now be obtained and made visible by automatic means. However, the visualization literature is still scarce on approaches that allow an insightful close and distant reading of the rhythmical patterns in a poetry corpus. In this work, we report our initial efforts in characterizing of the visualization design space of distant and close reading of poetic rhythm. By employing a digital version of a corpus of 11,268 verses originally written by the Spanish poet and playwright Federico García-Lorca (1898–1936), we could craft several prototypical visualizations representative of the inherent complexity of the problem which we expect to employ in future user studies and that we share here with the rest of the community to foster further discussion around this interesting topic.

## 1. Introduction

Poetry is a special form of literature that employs language in unusual ways to evoke emotion, paint vivid imagery, or convey a message in musical and imaginative ways, and it can draw intense emotion out of readers in manners that regular prose cannot (Abdul-Rahman et al., 2013). To do so, imagery, metaphor, rhyme, meter, and other literary devices are employed to convey meaning and aesthetics in pieces of structured or semi-structured text named stanzas, which are combined into large pieces known as poems. Poetry contains a high density of creativity and ambiguity and it encompasses a wide range of forms, styles, and themes, such as politics, love, or the natural world, to name a few.

In the field of computer-based automated poetry analysis, a method focus on stylometry, which involves measuring statistical features such as the frequency of words or syllables or entropy. This is done to compare styles across groups of works from different cultural movements, periods, or places, in order to uncover recurrent patterns, uses of language, or expressions that can support a critical analysis of the works. The process of inquiry in stylometry follows a typical data analysis workflow, very similar to the traditional data analysis process of experimental sciences, in which data is collected, pre-processed, analyzed, and finally interpreted (Hullman and Gelman, 2021). This process typically starts with data aggregation according to a set of features of interest, followed by computing statistics of interest for each group, and finally establishing a comparison between the statistics of different groups to identify significant similarities and differences that were found, such as the recurrent use of certain words in this case. Among the typical features employed in stylometric analysis of poetry, we can find verse and poem length, rhyme, use of words and parts of speech, or semantics. However, the analysis of sonic and rhythmical patterns is exclusive to poetry, as it cannot be found in any other kind of literary work.

Poetry scansion refers to the analysis of the rhythmic structure of a poem and involves recognizing and counting specific patterns in a poem, such as the stressed and unstressed syllables that can be found (Agirrezabal, 2017; Navarro-Colorado, 2018). Although scansion is intimately related to syllabification through the prosodic features of the language in question, these two terms should not be conflated, as syllabification refers to the mere splitting of words into their constituent units, the syllables, according to the general rules of a language. Thus, a successful scansion typically depends on the accurate identification of prosodic features, such as stress and lexical tone, which are first obtained through the syllabification of the poem's verses (Navarro-Colorado, 2015; Agirrezabal et al., 2017). On its own, scansion is a useful technique for determining the form and style of poetry from different eras and cultures, and it is widely accepted as a keystone scholarly practice in the field of literary analysis. However, the process of scansion is rather complex and has traditionally been done manually in a highly time-consuming manner. Luckily, recent breakthroughs in automatic text processing have resulted in a surge of language-specific computer-based scansion methods (Agirrezabal, 2017; Navarro-Colorado, 2018; de la Rosa et al., 2020), opening up new exploration avenues for digital poetry corpora in many of the most popular languages of the world (Agirrezabal et al., 2016).

This paper presents a conceptualization of the under-explored (Benito-Santos and Therón Sánchez, 2020) visualization design space of distant and close reading^1^ of poetical rhythm, which is tested on a corpus of sonnets by the Spanish poet Federico García-Lorca (1898-1936). Spanish poetry, due to the somewhat rigid accentuation rules of the language “meaning that graphemes and stress can be directly derived from written words using a simple set of rules”, historically has been a good candidate to exemplify scansion algorithms (Agirrezabal, 2017; de la Rosa et al., 2020).

Due to the unavailability of computational methods to produce an automatic scansion of the poems, previous approaches focused on the close reading of the rhythm in manually annotated poems (Abdul-Rahman et al., 2013; McCurdy et al., 2016), or on a distant reading of other features different from rhythm (e.g., semantic) found in a collection of poems (Madnani, 2005; Delmonte, 2015). Thus, ours is the first attempt to produce a visualization scheme that can be scaled to visualize the rhythm of one or many poems employing a completely automatic approach. This was facilitated by our active involvement in trying to make text a “first-class attribute” (Brath, 2018, 2020) in our proposed visualizations, meaning that we recurrently exploit some of its visual attributes to encode data features. This is done through a series of prototypical designs based on the heatmap visualization whose aim is to provide a first approach to the problem of visualizing poetic rhythm, and to drive future user studies that are more aligned with the reality of the problem.

## 2. Corpus

**Figure 1:**
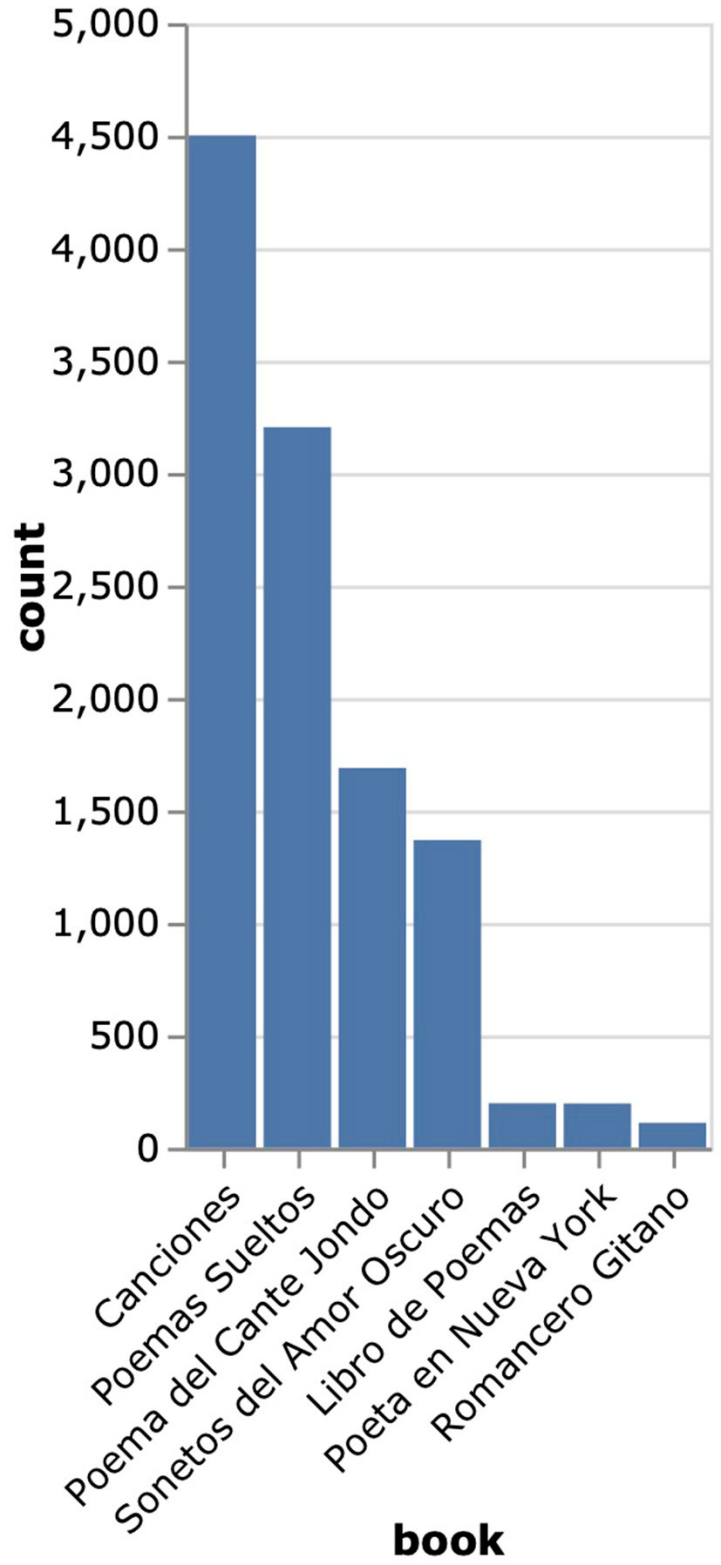
Distribution of the number of verses by book in García-Lorca's poetic corpus.

To test our designs, we selected a complete poetic corpus of the Spanish poet, playwright and theater director Federico Garc-a-Lorca^2^, which we compiled from the website https://federicogarcialorca.net/. The corpus contains 225 poems (11,268 verses) from 7 different books and compilations, encompassing García-Lorca's main poetic works from the 1920s until his death in 1936 (see Figure 1).

We employed the Python library *Rantanplan* (de la Rosa et al., 2020)^3^ to obtain an automatic scansion of all the poems in the corpus. The library employs a fast, rule-based method to syllabify the words in a poem, and, using several heuristics, tries to adjust the verse to known rhythmical structures of Spanish poetry. In their paper, the authors claim that it obtains accuracy scores of 99.99% on the syllabification task and 92.75% on the scansion task in a corpus of 10,000 annotated verses of sonnets “a sonnet contains 14 hendecasyllable verses” from the Spanish Golden Age (1492–1659). However, the authors also report an important decay in performance when analyzing a collection of 4,300 mixed-meter verses (65.02%). Despite our corpus holds poems of very different structure, we considered the library's performance good enough to support a first exploration of our corpus, an endeavor that has already unveiled some potential lines of enhancement for *Rantanplan*.

After successful processing of a poem, *Rantanplan* outputs a list of strings “one per verse” of plus “+” and minus “−” symbols, denoting strong “stressed” and weak “unstressed” syllables (e.g., “–+-+-+-”). These sequences, along with other metadata such as the poem's title, or the collection it belongs to, were stored in JSON format^4^ and visualized in the statistical visualization library Altair (VanderPlas et al., 2018), which was employed to build all designs presented in this paper.

## 3. Design process

### 3.1. Design considerations

Typically, when dealing with text as the main data source in a visualization project, the most important entities of the text are extracted, summarized, and ultimately represented as symbols as if they belonged to data of any other different nature. For example, to build a word cloud it is first necessary to compute a statistic (typically the frequency or another centrality measure) that is used to drive the final size and position of the words (Jänicke et al., 2017). However, much information is lost in this process: for example, the original order of the words can no longer be grasped from the visualization. Although several techniques try to overcome this issue, typically through interaction, they also inevitably impose an important burden on the user, who has to constantly alter his focal point of attention, with all the unwanted consequences that this entails. In addition, much of the reticence of the humanities community to incorporate visualization systems into their traditional workflows is partially rooted precisely in this loss of information, which is a major current challenge in the discipline (Drucker, 2011; Therón Sánchez et al., 2019; Panagiotidou et al., 2023). Also, a major hurdle in designing text-based visualizations is that text is not preattentive: it must be read (in Occidental countries) from top to bottom, from left to right and word by word, to understand its meaning in a slow process compared to the typical forms of visual reasoning (Brath, 2018). However, this disadvantage is also the reason for its vast communicative power: written text is one of the major technological inventions of the human race, as it allows us to communicate complex thoughts, ideas, and sentiments to other people in other places and times with an incredibly low amount of resources.

Moreover, poetic texts are shorter and pay more attention to structure than other forms of literature. This, added to the fact that text and rhythm are intimately related in poetic compositions “as the second emerges directly from the first”, the simple depiction of summaries is much less interesting in this case. For these reasons, we consider poetry visualization a special case within the larger discipline of visualization for the humanities because it requires that we craft visualizations and interaction patterns that feel even “closer” to the text, effectively reducing the effort that is required to arrive at a close reading of a piece.

**Figure 2:**
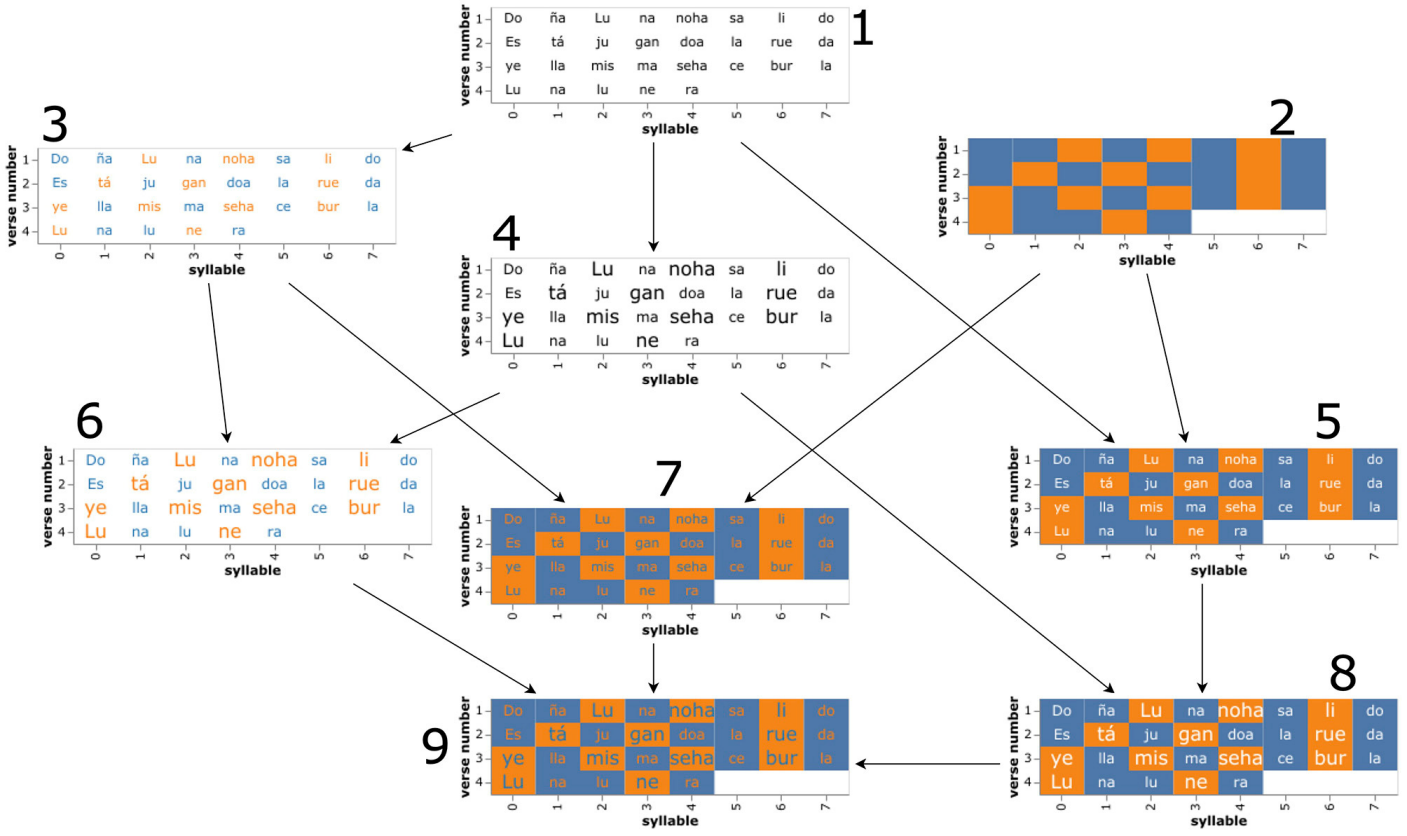
Evolution of different approaches to close reading a poem with rhythm and text. The poem is 1922 “Recuerdo” (“Memory”) by García-Lorca (see Section 4 for an English translation). The number of visual channels employed by each design can be derived by counting the number of arrows.

The upcoming sections detail the internal design process we followed to characterize the solution space of rhythm visualization. Spearheading this process was one of the coauthors of this paper who also is one of Rantanplan's creators, whose indications facilitated the development of the visualizations presented below. The design process adopted a systematic, bottom-up methodology, progressing from fundamental building blocks to more complex structures, which is discussed in detail in the following sections: in 3.2, a first set of visualizations aimed at the close reading of the poemts in the collection, and representing text and rhythm (shown in Figure 2) is introduced. Then, we describe how we kept overloading the base visualizations to arrive at a final design centered on the close reading task and consider different scenarios in which the final design we arrived at could be modified to support a distant reading of rhythm in the collection, covering from 1-to-1 comparisons (3.3) to visual aggregations of multiple items (3.4).

### 3.2. Close reading: visualizing one poem

Close reading is a fundamental task of literary scholarship which is key to create informed critical analyses of a body of literature (Jänicke et al., 2017). In this section, we describe the different steps that we took to create a prototypical joint visualization of text and space, commenting on the intermediate designs that we obtained along the process, which can be followed in Figure 2.

Two main features of the text that we aimed to display were the text and the rhythm. Initially, we propose two simple visualizations that served as the foundation for the rest of the designs that came after. These intermediate designs are listed below (on each list item, numbers between parentheses refer to the the intermediate designs of Figure 2).

- **Text (1)**: As a first approach to the problem, we simply displayed the poem's syllables in the plane, mapping the order of appearance of the verse in the poem to the y coordinate, and the position of the syllable in the verse to the x coordinate. With this visualization, the effects of the scansion process are already evident, for example in the first row, where it can be seen how the algorithm has joined two syllables from different words (“no” and “ha” into “noha”), a linguistic artifact known as synalepha.
- **Background color (2)**: It is the default option, where the text has been removed, and the rhythm is depicted as the cell's background where the syllable in question would have resided. Whereas if the purpose is to enable a close reading of the poem, a great amount of information is lost, this visualization allows the reader to focus completely on the rhythm. In addition, it allows comparing two poems side by side, estimate the amount of stress in a poem, and also could be used for visually aggregating poems (see 3.4). In the example, we use two categorical colors from the *category10* scheme to represent stress, or the lack of it, respectively. These base visualizations were modified by introducing redundancy in the other available channels that were considered in the experiment. We discuss them hereafter:
- **Text color (3)**: Another straightforward option to represent the stress in a verse is to use a color-coding approach. The idea is to assign one color for stressed and another for unstressed syllables, thus making it easy for the reader to identify them. In the figure, we employ the same color scheme that we used for the backgrounds. This approach is more neutral in terms of legibility and allows a good comparison of consecutive verses, but cannot be used to aggregate poems nor does it work well for establishing quantity estimations (Correll et al., 2011, 2013).
- **Text size (4)**: We also decided to test the visual channel of text size, as it is also paradigmatical for displaying emphasis and importance in a piece of text (Brath, 2018). In this case, we employ a linear scale to map the dichotomous variable stress to two different values of font size. The font size will have to be carefully picked, given that if the difference is too large, it will allow for an easy distinction of stressed/unstressed syllables in prejudice of legibility and vice-versa, should the difference be too small. Additionally, this ratio could be a function of the poem's size, in a way that for longer poems the difference ratio is decreased to allow for a more rapid inspection of the whole poem. For shorter poems, this would not be necessary and a large ratio, allowing a finer comparison, might be a better option. In any case, the text size ratio property is a good candidate to be controlled through *ad-hoc* interactive controls that the user can manipulate at his or her discretion. In our initial tests, we used a size ratio of 1.33 between stressed and unstressed syllables.
- **Background color + Text (5)**: In this case, we combined cell background color with the verse text. Here, the text base color was changed from black to white to have better contrast with the background and increase its legibility. This visualization keeps the beneficial properties of approach 2 of the previous section but also adds textual information. In addition, the text could be hidden and displayed using interactive controls, allowing users to focus solely on the rhythm at their own discretion. Natural extensions of this representation involve adding text size and text color, which are presented below.
- **Text color + Text size (6)**: In this combination, stress is represented as text color and text size, which seems to better convey the information than the single-channel versions of its components. However, visual quantity estimation is degraded in favor of better legibility.
- **Background color + Text color (7)**: Despite maintaining a small text size and a good contrast with the background, the quantity estimation capabilities of this design are negatively affected by the text color, making it less apt for distant reading tasks.
- **Background color + Text size (8)**: This mixture allows for a more rigorous comparison of consecutive verses and makes the legibility of the text high, which makes it a good candidate for close reading tasks. In addition, and due to its implementation of background color, it should be efficient in distant reading tasks such as visual aggregation or quantity estimation.
- **Background color + Text color + Text size (9)**: Taking the ergonomics of reading into account, the text could be colored by inverting the color scale used for the background (see bottom of Figure 2), producing similar effects to the previous situation but using less brightness which could be more suitable for long reading sessions. However, due to the mixture of colors, the capacity of estimating the total number of stressed syllables in the poem seems to be negatively affected, meaning that overally this design behaves worse than its white-text counterpart (number 8).

### 3.3. Comparing the rhythms of two poems

When we considered that we had arrived to a functional design for the close reading task, we used this design to evaluate its capabilities to enable a side-by-side visual comparison of the rhythms of two poems, which it may interesting for an analyst for several reasons (e.g., stylometric comparison between the same or different authors, authorship attribution, etc.). Here, we reasoned that the simplest approach would involve adding the verses of the second poem to the visualization of the first one. For example, when comparing quatrains, two verses per “y” coordinate would be shown for a total of eight verses displayed. Additionally, to make the separation between verse lines clearer, a small gap between the four rows could be introduced. In our preliminary tests, the combination of background color and text size also seemed to work well too in this context, although we aim to corroborate this first impression in an experimental setting with real users.

### 3.4. Distant reading of rhythms

**Figure 3:**
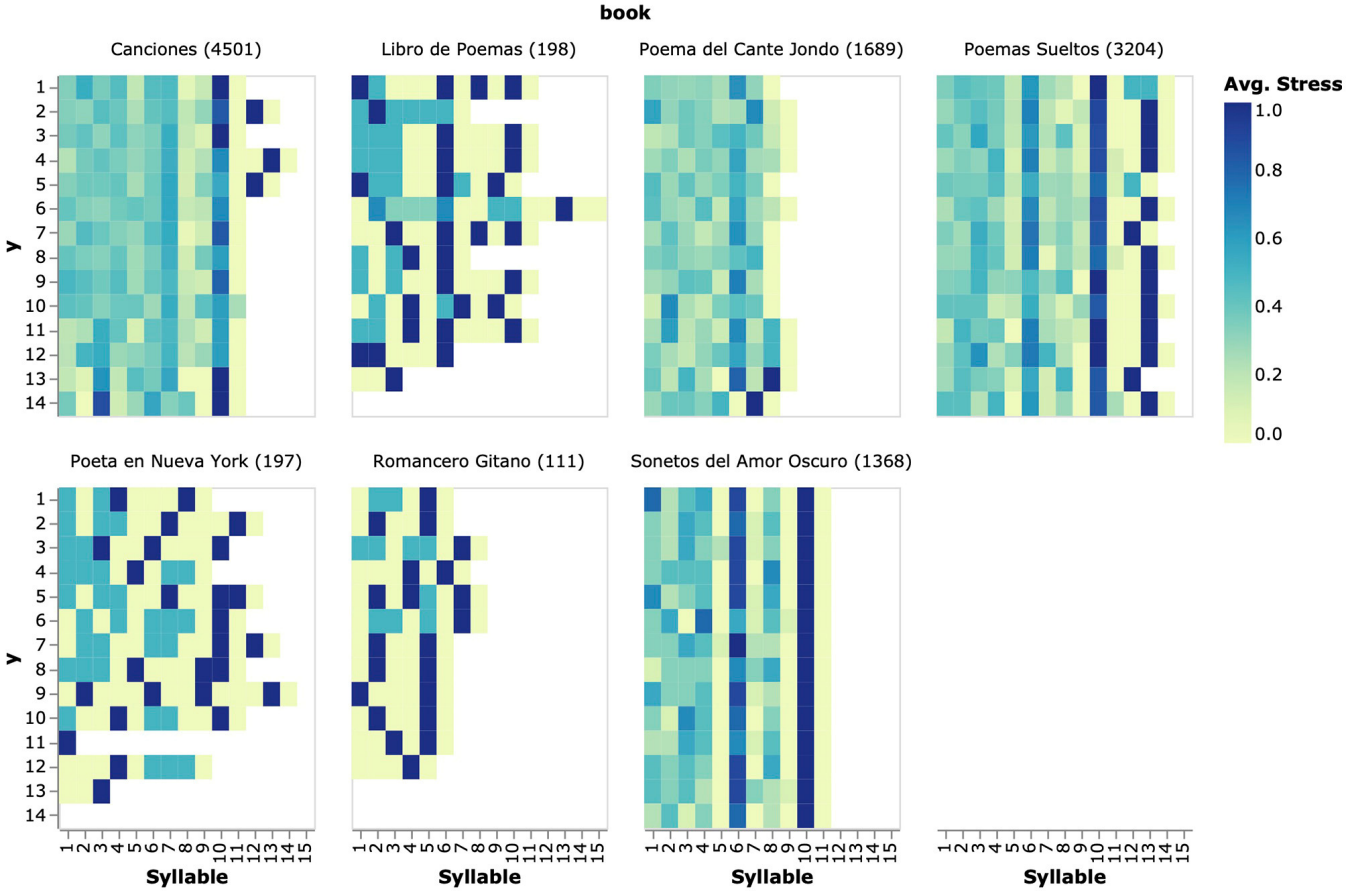
Seven heatmap visualizations showing poem structure and rhythmical patterns across all books included in García-Lorca's poetic corpus. At the top of each heatmap, the title of the book and the number of verses are displayed. A recurrent practice in metrical analysis involves aggregating data by a certain feature of interest, which is demonstrated here by extending the single-book visualization of the preceding section to compare rhythmical patterns across all verses in the corpus.

Figure 3 shows a heatmap displaying the average stress calculated per book, verse number, and syllable. Apart from differences in poem and verse lengths, the chart displays a mixture of the author's adherence to traditional Spanish poetry standards and also his own preferences and human creative genius. In the figure, the structure of several poetic artifacts can be appreciated. For example, the rules of the Spanish sonnet establish that this poetical form must be composed of fourteen hendecasyllables (11-syllable long verses).

Regarding accentuation, the rules state that it is obligatory to place accents in the 10th “penultimate” and 6th or in the 10th and 4th, and 8th syllables of each verse. This effect is depicted on the heatmap of the figure representing the 1,368 verses in the sonnet compilation “Sonetos del Amor Oscuro” (“Sonnets of Dark Love”), which also shows Garcia-Lorca's preference for composing verses that place the stress in the 6th and the 11th syllables. The heatmap also conveys the variability in the initial “first 4” syllables of the verses in this book, which is possibly due to a relaxation of the accentuation rules in the first syllables of the sonnets' verses. This is an important fact to notice by a literary scholar, since it reveals the author's creative experimentation with the poetic form, which is also captured by this design.

Also, in the same manner that it is important to identify structural patterns, it also might be interesting to make the lack of it more obvious. This typically happens when an author writes a poem with no clear meter or structure, as is often the case in free verse works, or when, as is the case, the proposed aggregation contains heterogeneous data. Unsurprisingly, this can be observed in the books containing the largest numbers of verses: “Canciones,” (“Songs”), “Poema del Cante Jondo,” (“Poem of the Deep Song”), and “Poemas Sueltos” (“Spare Poems”). All these collections include a variety of mixed-meter poems that would require further inspection by an analyst “for example by trying to categorize the poems by matching them to a set of traditional types of stanza,” enabling an analysis exercise of fixed-meter poems similar to the one we performed on “Sonnets of Dark Love.”

**Figure 4:**
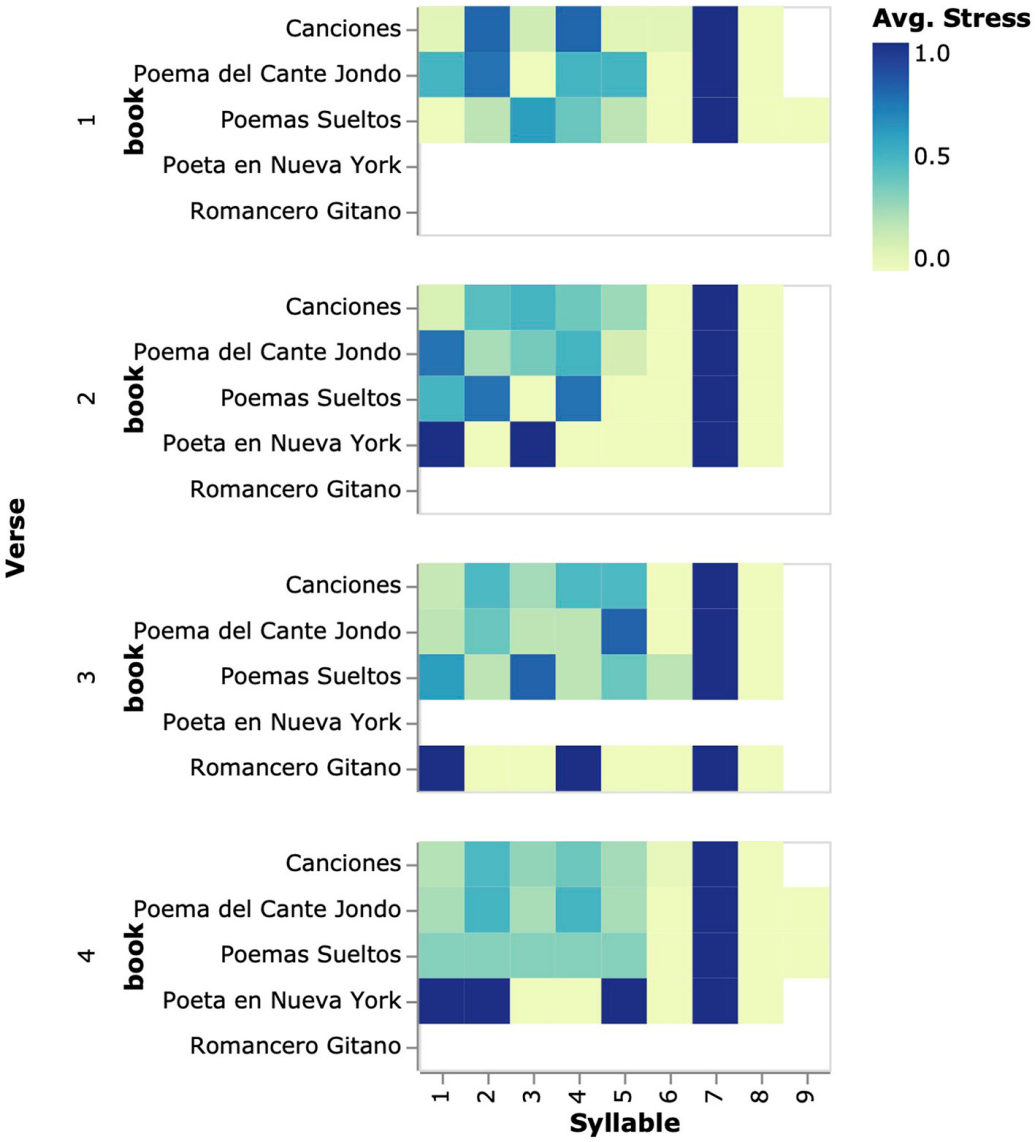
By modifying previous designs, this visualization allows the comparison of rhythmical patterns across verses placed at the same line numbers in different poems. In the example, only verses of length 8 appearing up to the 4th position in the poem are shown. The chart shows a clear tendency to stress the penultimate syllable.

To allow for a finer inter-poem comparison of verses, we aggregate them by the ordinal in which they appear in the poem they belong to and present them in a similar heatmap to those of previous sections but with the exception that in this case we use text again to indicate the number of data points. Two aspects are worth discussing from the chart of Figure 4: firstly, all 936 verses place the stress in the 7th syllable regardless of the collection they belong to, or their position in the poem. The chart also shows which collections have missing data (i.e., the categories are not removed from the chart when they have no data, which would be considered bad practice). For example, “Romancero Gitano” (“Gypsy Ballads”) does not contain 8-syllables-long verses in the 1st, 2nd, and 4th positions, “pointing to higher variability in verse length,” and only one verse in the 3rd line can be seen displaying a distinctive rhythmical pattern of the book (c.f. Figure 3).

## 4. Conclusion and future work

In this study, we set out to characterize the visualization design space of poetic rhythm through a series of exploratory designs. These initial experiments hint at the exciting research opportunities that emerge from the fusion of automatic poetry analysis and visualization, a highly underexplored area that we believe deserves more attention from the visualization community. Despite these initial insights, the presented visualizations also show important shortcomings related to the loss of information that occurs during the aggregation process. In particular, the visualization does not inform about the particular distribution of values on each syllable, hiding much of the variability and richness that lies in the data, a practice that many authors in visualization and the humanities have argued against in the past (Drucker, 2011; Coles, 2016; Therón Sánchez et al., 2019; Windhager et al., 2019). As a consequence, it is difficult to infer confidence intervals around the depicted statistic (i.e., the proportion of stressed syllables). In this paper, we added text (as seen in Figure 4) to overcome this issue but this solution does not take into account the empirical distribution of values around the mean, obscuring important information that is known to be error-prone (Hullman and Gelman, 2021). Thus, new designs that take into account these considerations are necessary to maximize the robustness of the proposed analysis workflows that motivate realistic critical analyses of literary corpora.

In addition to the visualization of uncertainty, during the development of the prototype visualizations, we noticed several other research opportunities that we believe are worth exploring, such as the integration of sonification techniques (Rönnberg, 2021; Aigner et al., 2022) that could help users grasp the poems' rhythmical patterns more efficiently by motivating an augmented reading of the poems in the corpus, with numerous potential benefits (e.g., for didactic purposes, or the creation of derived works).

Beyond these considerations, our prototypes also fail at capturing the important role combinatorics play in the composition of poetic literature (Poibeau et al., 2020). To illustrate this concept, take the example of the accentuation rules of the sonnet. Constraining the placement of the stress at the penultimate syllable leaves the writer with considerably fewer options (word combinations) to create a verse that complies with the said constraint. The inclusion of this kind of elements in the visualization could lead to “what-if” types of pattern analysis in which other possible alternative versions of a poem, employing other word combinations and rhythmical patterns, are explored (Wang et al., 2017). This, combined with the incorporation of elements related to rhyme or semantics, could have important implications in the field of literary analysis, enhancing a wide range of scholarly tasks ranging from traditional critical practices to creative writing and composition. To validate these hypotheses, we are organizing a series of workshops with experts in literary studies within the context of the European project CLS Infra (https://clsinfra.io/), from which we expect to recruit a large user base for driving the studies to come. In this regard, we want to extend an open invitation to all members of the visualization and humanities communities interested in contributing to our research to join us in this exciting project.

## Data availability statement

The datasets presented in this study can be found in online repositories. The names of the repository/repositories and accession number(s) can be found in the article/supplementary material.

## Author contributions

The primary intellectual and creative contributions to this work were made by the lead author, who also drafted the manuscript. The additional authors provided valuable input through their proofreading efforts, and contributed crucial data. All authors reviewed and approved the final version for publication.

## Footnotes

^1^Close reading involves a meticulous analysis of a smaller portion of text (e.g., a single poem) focusing on details such as language, syntax, and structure, while distant reading enables the analysis of larger corpora of texts, revealing overarching patterns and trends.

^2^ https://en.wikipedia.org/wiki/Federico_Garc%C3%ADa_Lorca

^3^ https://rantanplan.readthedocs.io

^4^The corpus is available for download at https://raw.githubusercontent.com/ale0xb/visualizing-garcialorca/main/poems/poems.json.

## Appendix

### Annex I: Translation of “Recuerdo”

Lady Moon is still hidden,

playing ring around a wheel.

She mocks herself:

“Looney moon.”

García-Lorca, F. “Recuerdo,” in “Poemas Sueltos,” 1921.

Free translation by the authors
